# Aberrant glycosylation patterns as potential biomarkers for diagnosis and disease progression in bullous pemphigoid

**DOI:** 10.3389/fimmu.2025.1538126

**Published:** 2025-05-26

**Authors:** Haijun Miao, Jundan Yang, Yaxing Bai, Shengxian Shen, Xia Li, Lixin Yue, Gang Wang, Erle Dang

**Affiliations:** Department of Dermatology, Xijing Hospital, Fourth Military Medical University, Xi’an, Shaanxi, China

**Keywords:** bullous pemphigoid, glycosylation, sialic acid, fucose, lectin/glyco-antibody microarrays

## Abstract

**Objectives:**

Bullous pemphigoid (BP) is a prototypical autoimmune disease characterized by the production of autoantibodies against hemidesmosomal proteins BP180 and BP230. Aberrant glycosylation has emerged as a possible mechanism linked to the pathogenesis and progression of autoimmune diseases. However, the precise alterations in glycosylation in BP remain largely unknown. In this study, we explored the molecular mechanisms of abnormal glycosylation in BP pathogenesis.

**Methods:**

We systematically investigated the glycosylation changes in serum, blister fluid, and saliva from BP patients using lectin microarray assays and lectin-based enzyme-linked immunosorbent assays.

**Results:**

Our findings revealed increased glycosylation modifications of sialic acid, galactose, and fucose in serum proteins from BP patients, as well as enhanced fucosylation, galactosylation, and biantennary N-glycan glycosylation in blister fluid proteins. Notably, these abnormal modifications of monosaccharides correlated with the clinical indicators of BP. Furthermore, we observed that glycosylation patterns in saliva were associated with disease severity, suggesting their potential as valuable non-invasive diagnostic markers for BP.

**Conclusion:**

These discoveries indicate that aberrant glycosylation patterns may provide insights into the pathogenesis of BP and serve as potential biomarkers for diagnosing and monitoring the disease.

## 1 Introduction

Bullous pemphigoid (BP) represents the predominant autoimmune subepidermal blistering disorder, characterized by inflammatory erythema and tense blisters, often accompanied by intense pruritus, predominantly affecting the elderly population (1). The underlying pathogenesis primarily involves an autoimmune response targeting the hemidesmosomal antigens, BP180 and BP230, triggering immune cell activation and subsequent infiltration of inflammatory cells (2). However, the mere deposition of autoantibodies at the dermal-epithelial junction as immune complexes does not exclusively elicit consequential inflammation, as observed in some patients and animal models (3). Additionally, the presence of Immunoglobulin G (IgG) autoantibodies has been identified in the serum of healthy individuals and patients with asymptomatic autoimmune diseases (4). This indicates that the presence of autoantibodies alone does not guarantee the development of clinical symptoms. Therefore, it is important to explore other crucial factors that contribute to the onset and progression of the disease.

Glycosylation modification, a prevalent post-translational protein modification, constitutes a potent regulatory mechanism, operating at similar protein expression levels, that holds promise for unraveling the pathogenesis of autoimmune disease (5). Under physiological conditions, glycoconjugates perform various functions including protein folding, signal recognition and immune tolerance maintenance. Abnormal glycosylation modifications can act as neoantigens that are presented by antigen-presenting cells, triggering immune recognition through the detection of atypical glycosyl epitopes. This process disrupts the delicate balance between immune tolerance and immune recognition (6). Moreover, it is widely acknowledged that IgG antibodies lacking galactosylation are associated with inflammatory conditions, whereas galactosylated and sialylated IgG antibodies are associated with attenuated inflammatory responses (7–10). Aberrant glycosylation has been implicated in the pathophysiological processes underlying various autoimmune inflammatory diseases (6), including systemic lupus erythematosus (SLE) (11), immunoglobulin A nephropathy (IgAN) (12), inflammatory bowel disease (IBD) (13), and rheumatoid arthritis (RA). However, the glycosylation profiles and their pathogenic roles in BP remain largely unexplored.

The investigation of glycosylation in pathophysiological mechanisms has been hindered by the limitations of available research tools, resulting in slow progress (14). Lectin microarray is a highly promising tool for glycan research developed in recent years. This technology utilizes the specific recognition properties of lectins towards glycans, enabling short-term batch testing and comprehensive screening (15). In this study, we aimed to investigate glycosylation patterns in the serum, blister fluid, and saliva of BP patients and identify the general characteristics of glycosyl changes specific to BP by using lectin microarrays. Furthermore, lectin enzyme-linked immunosorbent assays (ELISA) were used to validate these changes in critical glycosyl structures and analyze their correlations with characteristics of BP progression. Finally, we assessed the alterations in abnormal glycosides in the saliva of BP patients before and after treatment, identifying the most relevant glycosides associated with disease changes. This comprehensive approach enables us to evaluate the potential of abnormal glycosyl structures as biomarkers for diagnosing and assessing the disease.

## 2 Material and methods

### 2.1 Patient recruitment and sample collection

33 serum samples, 32 blister fluid samples, and 23 saliva samples were collected from patients with BP at the Department of Dermatology, Xijing Hospital. Serum and blister fluid samples were obtained prior to treatment initiation (diagnostic phase) to ensure that baseline glycan profiles remained unaffected by therapeutic interventions. Among the saliva cohort, 18 received systemic glucocorticoids, 1 received topical halometasone due to complications, and 2 required adjunctive therapies (dupilumab or mycophenolate mofetil) for refractory disease; 3 were managed via outpatient care. Patient treatment regimens are detailed in **Supplementary Table 1**. BP diagnosis was based on typical clinical and histological presentation, direct or indirect immunofluorescence examination, and levels of the circulating autoantibodies against BP180-NC16A. 33 serum samples and 23 saliva samples, matched by age and sex, were chosen from the Medical Examination Center of Xijing Hospital. None of healthy controls (HC) presented with evidence of chronic autoimmune diseases or acute infections in their blood. 13 blister fluid samples generated during skin grafting were used as control samples for the BP blister fluid research section. All procedures involving the use of human samples were performed in accordance with the institutional guidelines and approval of the Ethical Committee of the Fourth Military Medical University, and were compliant with principles of the Declaration of Helsinki. Informed consent and permission to collect blood, blister fluid and saliva were obtained from all study participants.

### 2.2 Lectin/glyco-antibody microarrays

The manufacture of lectin microarrays and data acquisition were performed as described previously (16, 17). The sugar-binding specificities of the lectins are detailed in **Supplementary Table 2**. Briefly, lectins were purchased from Vector Laboratories (Burlingame, CA) and Sigma-Aldrich (St. Louis, MO) and dissolved into the manufacturer**’**s recommended solution at a concentration of 1 mg/mL, which contained 1 mM of the appropriate monosaccharide. Then, lectins were spotted onto homemade epoxysilane-coated slides, and each lectin was printed in triplicate. Microarrays were blocked using the blocking reagent (2% (w/v) BSA in PBST, 10 mM PBS buffer containing 0.02% (v/v) Tween-20) for 1 hour. The slides were washed three times and centrifuged. The extracted proteins from samples were labeled by Cy3 fluorescent dye (GE Healthcare, Biosciences, Piscataway, NJ, USA) and purified using a SephadexG25 column (GE Healthcare). Subsequently, 4 µg of labeled protein was mixed with 120 µL of lectin microarray incubation buffer and applied to the lectin microarrays and then incubated in the chamber at 37°C for 3 h. After washing three times, the slides were centrifuged to dry and scanned by using a GenePix 4000B confocal scanner (AXON, Instruments, Inc.). The fluorescence intensities were extracted by GenePix 6.0 software (Axon). To eliminate the influence of non-specific adsorption, the signal values less than average background + standard deviations (SD) were excluded from each data point, and global normalization was used to eliminate fluorescence bias. Demographic characteristics of the study participants whose samples were analyzed via lectin/glyco-antibody microarrays are summarized in **Supplementary Table 3**.

### 2.3 Lectin ELISA

Lectin ELISA was performed on serum, blister fluid and saliva samples. Briefly, during Lectin ELISA of serum and blister fluid, biotinylated lectins were diluted with 1% BSA to the following concentrations: HPA-L (Vector, B-1115) 5μg/mL, SNA (Vector, B-1305) 0.5μg/mL, PSA (Vector, B-1055) 0.5μg/mL, SBA (Vector, B-1015) 0.5μg/mL, RCA120 (Vector, B-1085) 0.05μg/mL, AAL (Vector, B-1395) 0.01μg/mL, while during Lectin ELISA of saliva, biotinylated lectins were diluted to the following concentrations: HPA-L 5μg/mL, SNA 2μg/mL, PSA 1μg/mL, SBA 5μg/mL, RCA120 1μg/mL, AAL 1μg/mL. Streptavidin linked to HRP (Vector, SA-5014) was diluted 1:2000 and incubated at 37°C for 30 minutes. The absorbance (optical density, OD) of each well was read at 450 nm by an automated microplate reader (Bio-Rad 680, Bio-Rad Laboratories, USA). Detailed demographic of the patients whose serum, blister fluid, and saliva samples were analyzed by lectin ELISA are provided in **Supplementary Tables 4–6**.

### 2.4 Anti-BP180-NC16A ELISA assay

The levels of anti-BP180-NC16A in saliva were measured using the BP180-NC16A ELISA kits (Medical and Biological Laboratories Co. Ltd., Japan). Following the manufacturer**’**s instructions, saliva samples were diluted at 1:101. A positive threshold (OD450 nm >0.066) was defined based on the upper limit of negative controls (healthy saliva samples), with positive controls derived from serum of confirmed BP patients. The absorbance (optical density, OD) of each well was read at 450 nm by an automated microplate reader (Bio-Rad 680, Bio-Rad Laboratories, USA). Detailed anti-BP180-NC16A ELISA results for all saliva samples are provided in **Supplementary Table 7**.

### 2.5 Statistical analysis

The data were presented as mean ± standard deviation or median ± interquartile range. Lectin microarray data were compared using an unpaired two-tailed t-test. Differences in lectin ELISA between two groups were assessed using the Wilcoxon test. Categorical variables were summarized as proportions and analyzed using the chi-square test. Correlation analysis was conducted using Spearman’s correlation test. All statistical analyses were performed using R, version 4.2.1, with the ‘rstatix’ package, version 0.7.2.

## 3 Results

### 3.1 Altered serum protein glycopattern of BP patients revealed by lectin microarray

**Figure 1:**
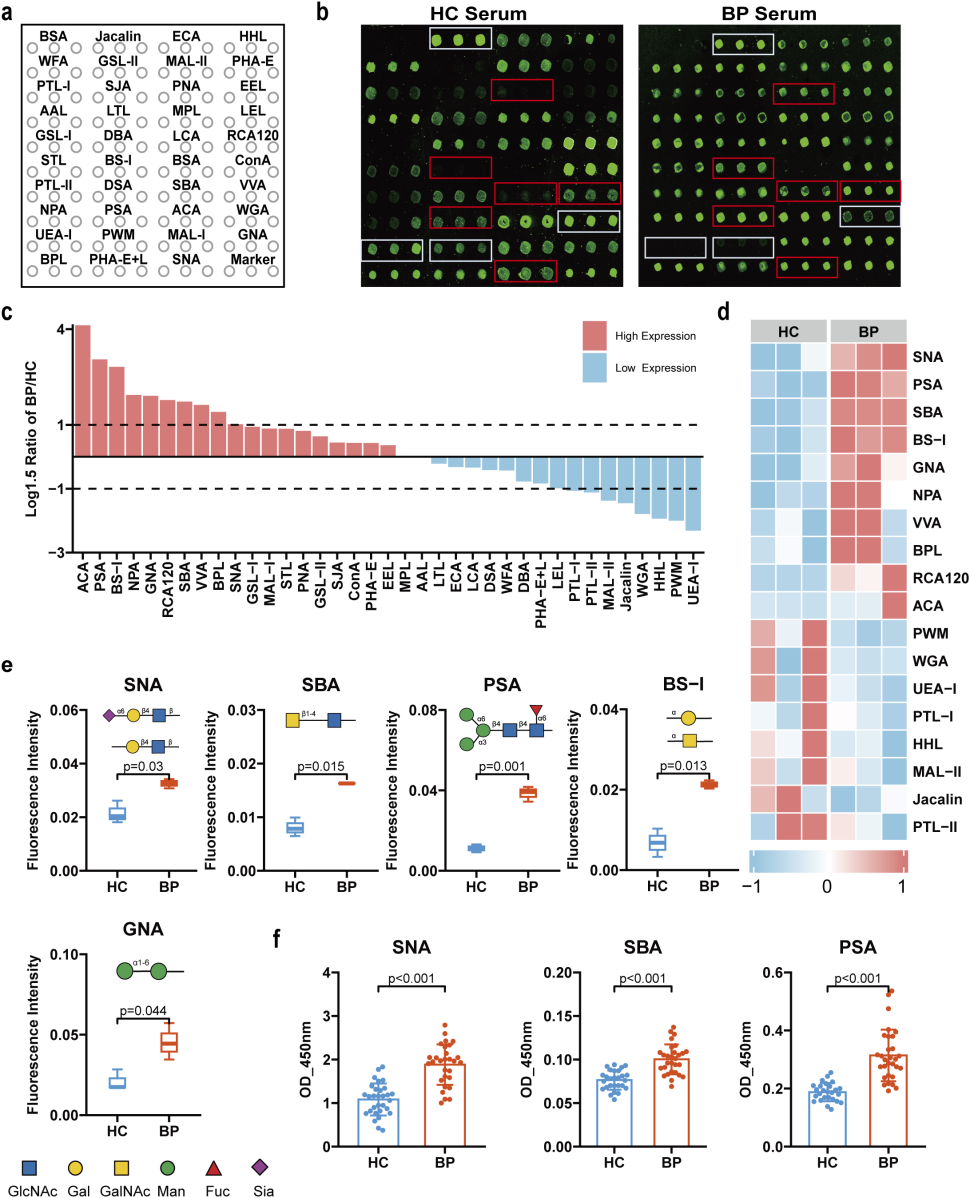
Alterations of serum glycopatterns in BP patients compared to healthy controls. **(a)** The layout of the lectin microarrays. A total of 37 lectins were uniformly distributed on the lectin microarray and each lectin was spotted in triplicate per block. **(b)** Representative results of the lectin array profiling for serum from BP patients (n=3) and healthy controls (n=3). Red boxes (increase) and white boxes (decrease) indicate the position of lectins with the significant differences between groups. **(c)** Comparative analysis of relative expression levels of 37 lectin-specific glycan structures in serum samples of BP patients compared to healthy controls. The ratio of the BP group to the HC group was logarithmically transformed to a base of 1.5. A ratio >1 indicates an increase of more than 1.5 times compared to the control group, whereas a ratio <-1 denotes a decrease of over 0.67 times compared to the control group. High expression is visualized in red, while low expression is depicted in blue. **(d)** Heat map of lectins with significant differentiation between BP patients and health controls. **(e)** Box plots showing the significantly altered lectins in serum of BP patients compared to healthy controls based on fold change. The data are presented as the averaged NFI ± SD of three biological replicates. **(f)** Lectin ELISA validated 3 lectins were significantly increased in serum of BP patients (n=30) compared to healthy controls (n=30). The data are presented as Mean ± SD. Analysis was performed using Wilcoxon Mann-Whitney test.

To decipher the glycosyl changes in BP, we profiled serum samples from 3 BP patients and 3 healthy controls using a lectin microarray that contained 37 lectins binding to specific glycans. The layout of the lectin microarray and representative profiling images are shown in **Figure 1a**. The result showed that 21 glycosyl structures exhibited increased expression, while 16 glycosyl structures showed decreased expression in the serum of BP patients (**Figures 1b, c**). Among all glycosyl changes, 18 key differentially glycans with a defined threshold (log_1.5_-fold change of the mean fluorescence intensity between BP and healthy >1 or <-1) were highlighted (**Figure 1d**) and we identified 5 glycans (**Figure 1e**) as the most significant differential glycans in BP serum. Compared with the healthy group, the serum protein glycosylation of BP patients presented unique changes: the monosaccharide levels of mannose (recognized by GNA, NPA, PSA), N-Acetylglucosamine (GlcNAc, recognized by PSA), N-Acetylgalactosamine (GalNAc, recognized by SNA, SBA, RCA120), galactose (recognized by SNA, BS-I), fucose (recognized by UEA-I).

To further validate the protein glycopattern analyses of BP serum as above, we performed lectin ELISA assay on serum samples from 30 BP patients and 30 age- and sex-matched healthy controls. The results revealed a significant increase of SNA (preferred Siaα2-6Gal/GalNAc residues, P<0.001), SBA (preferred α- or β-linked terminal GalNAc, (GalNAc)n, GalNAcα1-3Gal, P<0.001), PSA (preferred α-D-Man, Fucα-1,6GlcNAc, and α-D-Glc, P<0.001) in serum proteins derived from BP patients compared to the healthy control group (**Figure 1f**). These results demonstrated that the glycosylation of sialic acid, galactose and fucose was significantly increased in the serum proteins of BP patients.

### 3.2 Blister fluid fucose, galactose and bi-antennary N-glycans glycosylation were increased in BP patients

**Figure 2:**
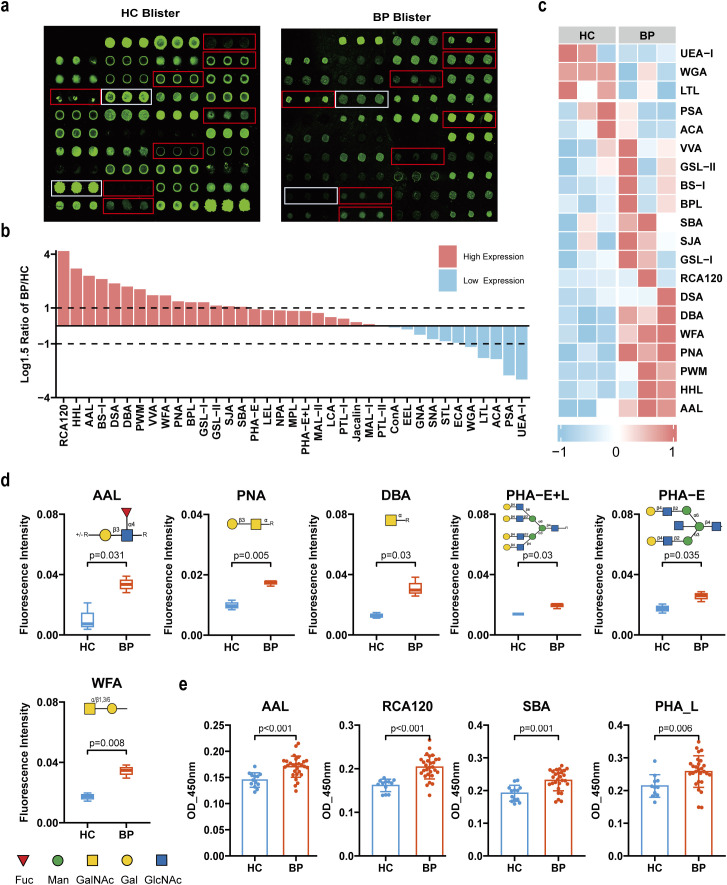
Alterations of glycopatterns in blister fluid of BP patients. **(a)** Representative results of the lectin array profiling for blister fluid from BP patients (n=3) and control samples (n=3). Red boxes (increase) and white boxes (decrease) indicate the position of lectins with the significant difference between groups. **(b)** Comparative analysis of relative expression levels of 37 lectin-specific glycan structures in blister fluid samples of BP patients compared to healthy controls. The ratio of the BP group to the control group was logarithmically transformed to a base of 1.5. A ratio >1 indicates an increase of more than 1.5 times compared to the control group, whereas a ratio <-1 denotes a decrease of over 0.67 times compared to the control group. High expression is depicted in red, while low expression is represented in blue. **(c)** Heat map of lectins with significant differentiation between BP patients and healthy controls. **(d)** Box plots showing the significantly altered lectins in blister fluid of BP patients compared to control samples based on fold change. The data are presented as the averaged NFI ± SD of three biological replicates. **(e)** The Lectin ELISA confirmed that 4 lectins were significantly increased in the blister fluid of BP patients (n=29) compared to control samples (n=13). The data are presented as Mean ± SD. Analysis was performed using Wilcoxon Mann-Whitney test.

Tension blisters serve as distinctive skin lesions in BP patients, with the fluid within these blisters being in direct contact with the diseased skin areas. We hypothesize that glycosylation modifications present in the blister fluid are more closely associated with the development of skin lesions. To gain a deeper understanding of the glycan profile characteristics in BP patients’ blister fluid, we collected samples from 3 BP patients, as well as 3 non-inflammatory blister fluid samples during skin transplantation, and performed lectin microarray experiments. Analysis of the lectin microarrays revealed the identification of 20 distinct glycans exhibiting differential expression patterns, as determined by a specific threshold (log1.5-fold change in mean fluorescence intensity compared to healthy controls, exceeding either 1 or -1) (**Figures 2a, b**). Specifically, 15 glycosyl structures demonstrated increased expression, while 5 exhibited decreased expression in the blister fluid of BP patients (**Figure 2c**). Among the various glycosyl modifications, glycans recognized by 6 lectins (AAL, PHA-E+L, PHA-E, WFA, PNA, DBA) have the most significant differences in BP blister fluid (P<0.05). Compared with the control group, the blister fluid protein glycosylation of BP patients presented unique changes: the monosaccharide levels of fucose (recognized by AAL), N-Acetylgalactosamine (GalNAc, recognized by WFA, NPA, DBA), galactose (recognized by WFA, PNA), GlcNAc (recognized by PHA-E+L, PHA-E) (**Figure 2d**).

To further validate the observed abnormal glycosylation in blister fluid proteins, a total of 29 patients with BP and 13 control blister samples were collected for lectin ELISA analysis. It was determined that blister fluid proteins derived from BP patients exhibited an increased presence of AAL (preferring Fucα1–6 GlcNAc (core fucose), Fucα1-3 (Galβ1-4) GlcNAc residues, p<0.001), RCA120 (preferring β-Gal, Galβ-1,4GlcNAc (type II), Galβ1-3GlcNAc (type I), p<0.001), SBA (preferring α- or β-linked terminal GalNAc, (GalNAc)n, GalNAcα1-3Gal, blood-group A antigen, p=0.001), as well as PHA-L (preferring GlcNAc, bi-antennary N-glycans, tri- and tetra-antennary complex-type N-glycan, p=0.006) (**Figure 2e**). These data suggest that the glycosylation of fucose, galactose, and bi-antennary N-glycans is significantly increased in the blister fluid proteins of BP patients.

### 3.3 The differential glycosylation patterns in both serum and blister fluid were correlated with clinical features and could serve as valuable diagnostic markers for BP

**Figure 3:**
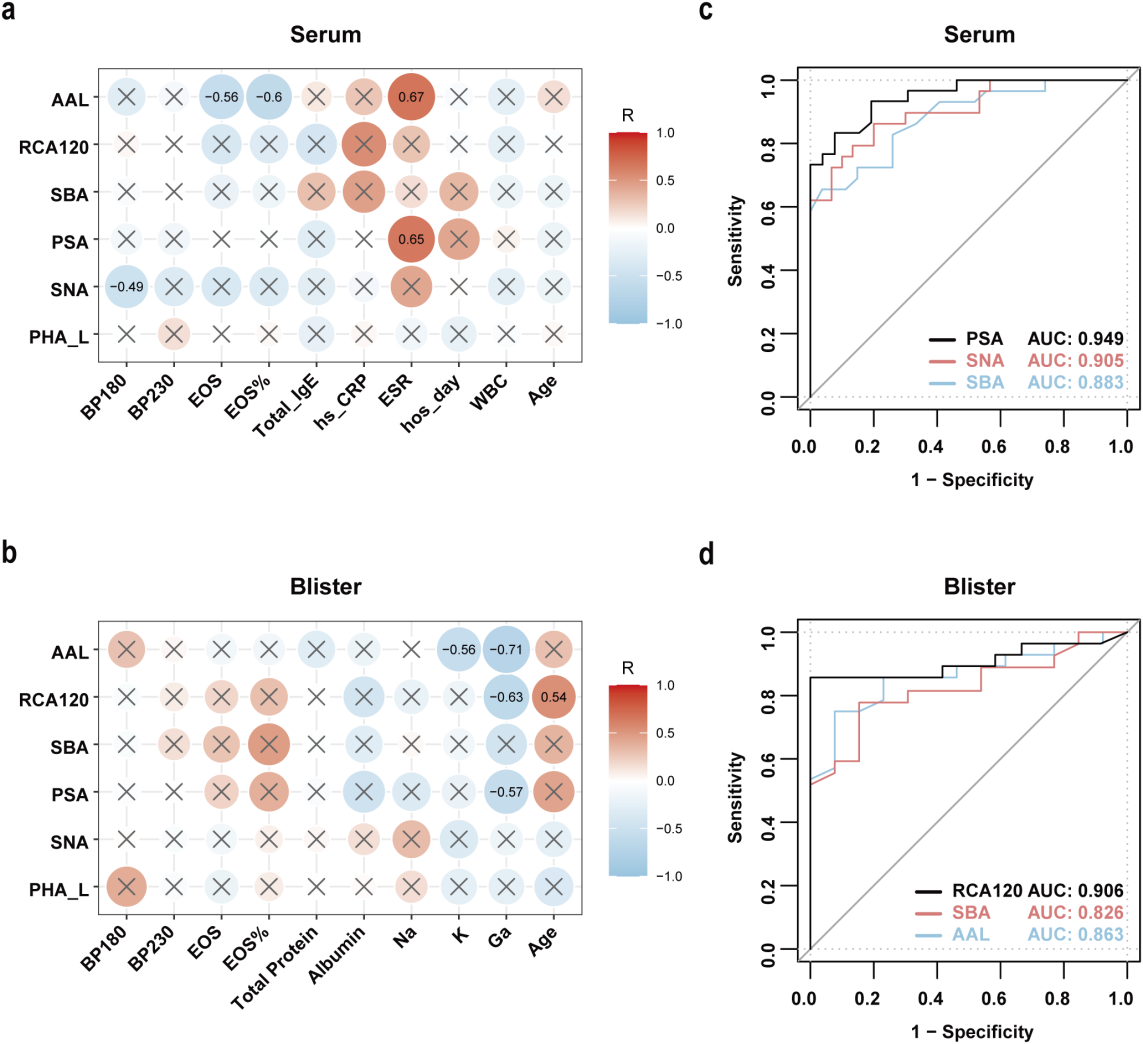
Correlation analysis of serum and blister fluid glycosylation with BP clinical indicators, and diagnostic accuracy assessed by ROC analysis. **(a)** Evaluation of the correlation between 6 glycosylation structures (binding to PHA-L, SNA, PSA, SBA, RCA120, AAL) and clinical indicators (BP180 and BP230 antibody titers, eosinophil count and percentage, white blood cell count, total serum IgE titer, erythrocyte sedimentation rate, high-sensitivity C-reactive protein level, hospital stay duration, age) in serum. **(b)** Evaluation of the correlation between 6 glycosylation structures (binding to PHA-L, SNA, PSA, SBA, RCA120, AAL) and clinical indicators (BP180 and BP230 antibody titers, eosinophil count and percentage, total protein, albumin, sodium, potassium, calcium ion concentrations, age, and the six glycosylation modifications) in blister fluid. Spearman’s correlation analysis was used for statistical testing. X indicates p>0.05 after statistical testing. Red color represents positive correlation, while blue color represents negative correlation. The larger and deeper the circle, the stronger the correlation coefficient. **(c)** ROC analysis for serum glycopatterns recognized by lectin PSA, SNA, SBA to discriminate BP patients from healthy controls. **(d)** ROC analysis for blister fluid glycopatterns recognized by lectin RCA120, SBA, AAL to discriminate BP patients from control group. The area under the curve (AUC) is used as a metric of diagnostic ability.

In order to identify the aforementioned abnormal glycans that are more closely associated with BP, we first collected clinical data corresponding to the serum samples, including BP180 and BP230 antibody titers, eosinophil count and percentage, white blood cell count, total serum IgE titer, high-sensitivity C-reactive protein level, and duration of hospitalization (detailed data information refer to **Supplementary Table 8**). After performing a correlation analysis, the results revealed that the glycan structures bound to SNA, AAL, and PSA in the serum were associated with the severity of BP. Specifically, SNA exhibited a negative correlation with BP180 antibody titers (r = -0.49, p < 0.05). AAL demonstrated a negative correlation with the percentage of eosinophils (r = -0.6, p < 0.05) and eosinophil count (r = -0.56, p < 0.05), while positively correlating with the erythrocyte sedimentation rate (ESR) (r = 0.67, p < 0.05). PSA also positively correlated with ESR (r = 0.65, p < 0.05) (**Figure 3a**). To further investigate the relationship between abnormal glycans in the blister fluid and the disease, we collected clinical data corresponding to the blister fluid samples, including BP180, BP230 antibody titers, eosinophil count and percentage, total protein, albumin, and particle concentrations (detailed data information provided in **Supplementary Table 8**). Correlation analysis revealed that the glycan structures bound to RCA120, PSA, and AAL in the BP blister fluid were associated with the disease. Among them, RCA120 positively correlated with age (r = 0.54, p < 0.05) and negatively correlated with Gallium particles (r = -0.63, p < 0.05). PSA negatively correlated with Gallium particles (r = -0.57, p < 0.05), while AAL negatively correlated with Potassium ions (r = -0.56, p < 0.05) and Gallium particles (r = -0.71, p < 0.05) (**Figure 3b**). These findings indicate that the glycan structures bound to SNA, AAL, PSA, and RCA120 are closely associated with the clinical features of BP.

Based on the close association between the aforementioned abnormal glycans and the disease, we posit that changes in glycans have potential value as diagnostic and predictive indicators of disease severity in BP. To further assess the diagnostic potential of the identified differential glycosylation in differentiating patients with BP from healthy individuals, receiver operating characteristic (ROC) curves were plotted using the logistic regression. The discriminatory performance of these glycosylation markers was evaluated using the area under the ROC curve (AUC). The results revealed significant discriminative ability in serum PSA, SNA and SBA with AUC values of 0.949, 0.905, and 0.883, respectively (**Figure 3c**). And blister fluid RCA120, SBA and AAL demonstrated favorable identification ability with AUC values of 0.906, 0.826, and 0.863, respectively (**Figure 3d**). These results indicated that altered glycosylation patterns in serum and blister fluid were correlated with disease severity and could serve as valuable diagnostic markers for BP.

### 3.4 Salivary proteins derived from patients with BP exhibited a higher abundance of terminal sialic acid, galactose, and fucose glycosylation

**Figure 4:**
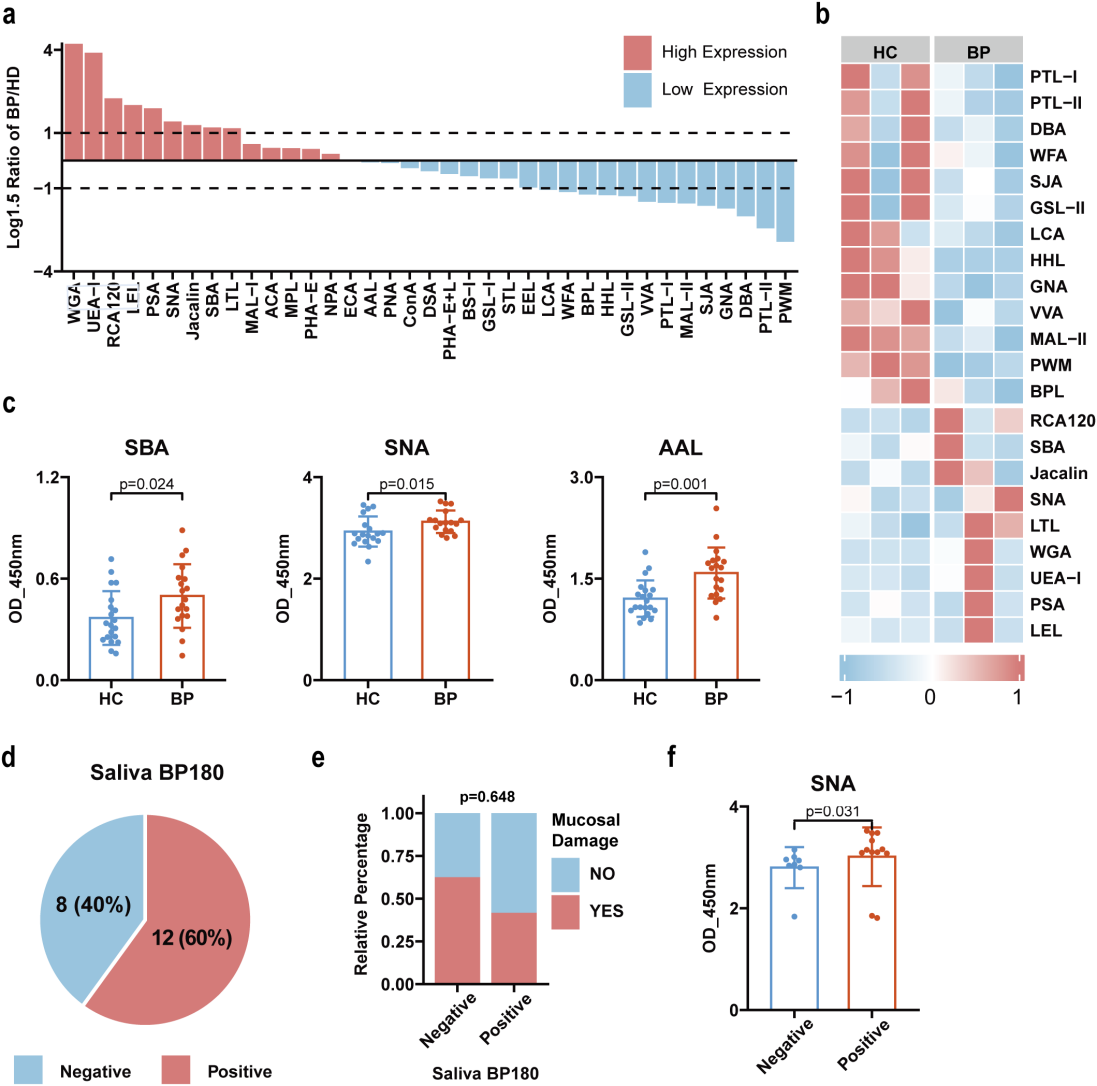
Alterations of glycopatterns in saliva of BP patients. **(a)** Comparative analysis of relative expression levels of 37 lectin-specific glycan structures in saliva samples of BP patients compared to healthy controls. The ratio of the BP group to the control group was logarithmically converted to a base of 1.5. A ratio >1 indicates an increase of more than 1.5 times compared to the control group, whereas a ratio <-1 denotes a decrease of over 0.67 times compared to the control group. High expression is depicted in red, while low expression is represented in blue. **(b)** Heat map of lectin with significant differentiation between BP patients and healthy controls. **(c)** The lectin ELISA confirmed that 3 glycans (binding to SNA, SBA, AAL) were significantly increased in the saliva of BP patients (n=20) compared to healthy controls (n=20). The data are presented as Mean ± SD. Analysis was performed using Wilcoxon Mann-Whitney test. **(d)** The ELISA was conducted to determine the positive rate of anti-BP180-NC16A antibodies in the saliva of BP patients. **(e)** Relationship between the presence of anti-BP180-NC16A antibodies in saliva and mucosal damage in BP patients. The data were presented as proportions, and statistical analysis was performed using chi-square test. **(f)** Differential analysis comparing the glycosylation structures (SNA) confirmed by lectin ELISA in the BP180 antibody-positive (n=12) and -negative groups (n=8) in saliva. The Wilcoxon Mann-Whitney test was used for statistical analysis.

As oral mucosa was the possible site affected in BP patients, and anti-BP180 antibody was detectable in saliva of some BP patients, we hypothesized that alterations in salivary protein glycosylation may be closely associated with the development of oral mucosal damage in BP patients. Saliva samples, collected from 3 BP patients and 3 healthy controls, were measured using lectin microarray assay. The results revealed that 9 glycosyl structures exhibited increased expression, while 13 glycosyl structures showed decreased expression in the saliva of BP patients based on the specific threshold (log1.5-fold change in mean fluorescence intensity between BP and healthy controls >1 or <-1) (**Figures 4a, b**). To further validate these changes in salivary glycosylation, we collected saliva samples from 20 BP patients and 20 age- and sex-matched healthy controls for lectin ELISA. The results showed that saliva derived from BP patients exhibited a significant increase of SNA (preferred Siaα2-6Gal/GalNAc residues, p=0.015), SBA (preferred α- or β-linked terminal GalNAc, (GalNAc)n, GalNAcα1-3Gal, p=0.024), and AAL(preferred Fucα1–6 GlcNAc (core fucose), Fucα1-3 (Galβ14) GlcNAc residues, p=0.001) in BP (**Figure 4c**). Subsequently, we measured the titers of anti-BP180-NC16A antibodies in the saliva of the 20 BP patients, wherein 12 (60%) patients tested positive for BP180-NC16A antibodies in saliva (serum positivity: 90%), compared to the negative controls (**Figure 4d**, **Supplementary Table 9**). However, there was no statistically significant disparity observed in relation to the proportion of mucosal damage between the salivary BP180-NC16A groups classified as positive and negative (p=0.648) (**Figure 4e**). Based on anti-BP180-NC16A antibodies in saliva, we further analyzed the glycosyl changes between salivary anti-BP180-NC16A antibody positive and negative patients. The results indicated that the lectin SNA (preferred Siaα2-6Gal/GalNAc glycosyl) exhibited significantly higher levels in the anti-BP180-NC16A antibody positive group compared to the negative group (p=0.031), while SBA and AAL showed no significant difference between the two groups (**Figure 4f**). These data suggested that sialic acid, galactose, and fucose recognized by lectin SNA, SBA and AAL were significantly increased in the salivary proteins of BP patients and sialic acid exhibit a much higher level in saliva anti-BP180-NC16A antibody positive patients.

### 3.5 Abnormal sialic acid and fucose glycosylation in saliva were correlated with disease severity and could serve as markers for monitoring disease progression

**Figure 5:**
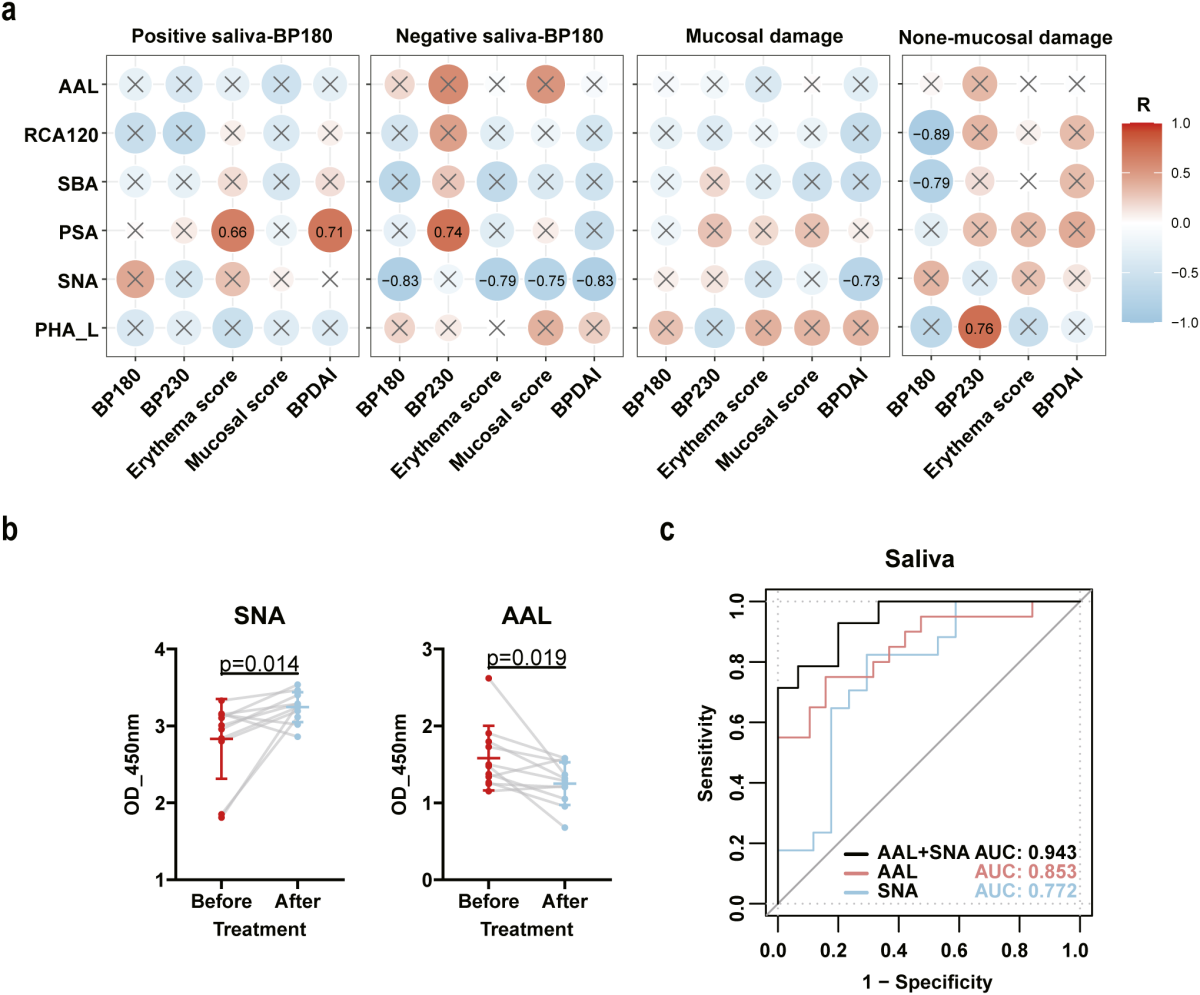
Correlation of salivary glycosylation with BP clinical indicators and diagnostic accuracy of selected lectins via ROC analysis. **(a)** Correlation analysis was conducted to identify the relationship between changes in six glycan structures (specifically binding to PHA-L, SNA, PSA, SBA, RCA120, and AAL) in saliva and disease severity indicators (BP180 antibody titer, BP230 antibody titer, erythema and wheal score, mucosal damage score, BPDAI score). BP patients were categorized into four subgroups based on the positivity of salivary anti-BP180-NC16A and the presence of oral mucosal damage. Spearman’s correlation analysis was used for statistical testing. X indicates p>0.05 after statistical testing. Red color represents positive correlation, while blue color represents negative correlation. The larger and deeper the circle, the stronger the correlation coefficient. The numerical values in the middle represent the Spearman correlation coefficients. **(b)** Changes in the glycan structures bound to SNA, and AAL in the saliva of BP patients were detected using lectin ELISA before treatment at admission and after significant improvement at discharge (n=11). The Wilcoxon Signed-Rank test was used for statistical analysis. **(c)** ROC analysis was conducted to assess the glycan structures bound to SNA and AAL in saliva as biomarkers. AUC was used as an indicator to evaluate diagnostic capability.

The clinical manifestations of BP patients exhibit heterogeneity, with some patients exhibiting mucosal involvement while others are limited to skin lesions. Our detection of BP180 antibody titers in saliva also revealed that not all BP patients have positive BP180 antibodies in their saliva, further illustrating the heterogeneity among BP patients. Based on the presence of BP180 antibodies in saliva and mucosal damage, we classified BP patients into four subgroups. Subsequently, we conducted a correlational analysis between six glycan structures (specifically binding to PHA-L, SNA, PSA, SBA, RCA120, and AAL) and five disease-related indicators (BP180 antibody titers, BP230 antibody titers, BPDAI score, mucosal score, and erythema score, detailed data information refer to **Supplementary Table 8**). The results indicated that in the saliva anti-BP180-NC16A positive group, the structures binding to PSA positively correlated with erythema score (r=0.66, p<0.05) and BPDAI score (r=0.71, p<0.05), suggesting that abnormally elevated fucose levels may contribute to more severe disease in patients with positive saliva antibodies. In the saliva anti-BP180-NC16A negative group, glycans binding to SNA negatively correlated with BP180 (r=-0.83, p<0.05), erythema score (r=-0.79, p<0.05), mucosal score (r=-0.75, p<0.05), and BPDAI (r=-0.83, p<0.05), while PSA positively correlated with BP230 (r=0.74, p<0.05). This suggests that decreased sialic acid and increased fucose in saliva may lead to more severe disease in patients with negative saliva antibodies. Among patients with mucosal damage, SNA negatively correlated with BPDAI score (r=-0.73, p<0.05), further indicating that reduced sialic acid is associated with more severe clinical symptoms in patients with mucosal involvement. In patients without mucosal damage, PHA-L positively correlated with BP230 (r=0.76, p<0.05), while RCA120 and AAL negatively correlated with BP180 (r=-0.89, r=-0.79, p<0.05). This suggests that abnormally elevated branched tri-mannose and reduced galactose are associated with more severe disease in patients without mucosal damage (**Figure 5a**). In light of the data, we can not only observe that abnormal glycosylation in saliva is closely related to the severity of BP, but also that different subgroups exhibit distinct glycosylation patterns associated with disease severity. This may be one of the reasons for the heterogeneity observed among BP patients.

To further assess the dynamic changes associated with abnormal glycosylation structures and disease states, we collected salivary samples from 11 patients with BP at the time of hospital admission prior to treatment and upon significant improvement at discharge. Through paired differential analysis, we observed an increase in the SNA-binding glycan structures (p=0.014) and a decrease in the AAL-binding glycan structures (p=0.019) in saliva compared to pretreatment levels. No significant changes were observed in the glycan structures bound by other lectins (**Figure 5b**). Comparative analysis between post-treatment patients and healthy controls revealed persistently elevated SNA signals (p=0.003) but normalized AAL levels (p>0.05, **Supplementary Figure 1**). These findings suggest that the glycan structures recognized by SNA and AAL exhibit high sensitivity as indicators of changes in BP disease status. Subsequently, we utilized the ELISA data of the two aforementioned lectins to perform a ROC curve analysis. The results demonstrated that both SNA and AAL exhibited good discriminative capabilities, with AUC values of 0.772 and 0.853, respectively. Furthermore, the combination of these two lectins significantly improved the discriminative ability, achieving an AUC of 0.943 (**Figure 5c**). These results suggest that altered glycosylation patterns in saliva are correlated with disease severity and could serve as non-invasive biomarkers for diagnosing and reflecting severity and prognosis of BP.

## 4 Discussion

Our study provides a comprehensive description of the characteristics of the glycopatterns in serum, blister fluid, and saliva of patients with BP. we observed profound changes in the glycosylation of serum, blister and saliva proteins in BP patients, indicating that glycosylation is closely involved in the development and progression of BP. Correlation analysis revealed that abnormal glycosylation is associated with multiple clinical indicators of the disease, suggesting a close relationship between glycosylation and the clinical presentation of BP. These characteristic changes in glycosylation form a unique glycoprotein profile in BP that has strong diagnostic ability and can serve as a potential diagnostic biomarker for the disease.

Glycosylation modification is a fundamental post-translational modification that significantly affects protein function and stability. Increasing evidence from recent studies has highlighted the disruptive effects of abnormal glycosylation modifications on protein performance and durability, thereby impacting cellular behavior. These modifications can lead to abnormal activation, migration, and signaling of immune cells, exacerbating the progression of autoimmune diseases. Abnormal glycosylation modifications exhibit high specificity and sensitivity, making them promising biomarkers for disease diagnosis. For instance, in rheumatoid arthritis, IgG galactosylation has been identified as a marker for distinguishing diseased individuals from healthy controls, while N-acetylglucosamine can differentiate osteoarthritis from rheumatoid arthritis (18–20). The detection of these specific glycosylation modifications allows for early diagnosis and precise typing of diseases. Furthermore, abnormal glycosylation modifications offer profound insights into disease prognosis and the effectiveness of treatment strategies. In this study, we conducted a systematic investigation of abnormal glycosylation pattern in BP patients. To the best of our knowledge, this is the first comprehensive analysis of glycosylation modifications in bodily fluids of BP patients. Our findings revealed that GlcNAc, galactose and fucose glycosylation were increased in serum or blister fluid of BP patient and correlated with disease progress. Importantly, the AUC of GlcNAc, galactose and fucose glycosylation recognized by PSA, SNA, SBA lectins in serum and RCA120, SBA, AAL lectins in blister fluid ranged from 0.826 to 0.949. These results highlight the high diagnostic value of glycosylation variations for BP.

The glycan profile of biofluids is shaped by both protein-specific glycosylation and the relative abundance of glycoproteins. While glycans on individual proteins exhibit tightly regulated, site-specific modifications, the total glycome of biofluids represents a composite of all glycoproteins present. In healthy individuals, glycan profiles demonstrate remarkable stability over time, due to the strong genetic regulation of glycosylation enzymes. This stability is evidenced by plasma N-glycan studies, which show minimal intra-individual variation (coefficient of variation: 5.6%) over periods ranging from days to years (21). In disease states, glycan alterations manifest as stable dysregulated patterns—persistent abnormal baseline levels that diverge from healthy profiles and have been utilized as biomarkers in various pathologies. Although inter-individual heterogeneity complicates the establishment of universal diagnostic thresholds, serial monitoring within the same patients can resolve disease-specific dynamics. This is particularly critical in BP, where long-term glucocorticoid management lacks objective biomarkers to guide initial dosing, maintenance duration, tapering schedules, and relapse prediction. Our findings demonstrate that SNA- and AAL-reactive glycans in saliva dynamically track disease activity and respond to therapy. Continuous assessment of these lectin signals could enable personalized management by detecting subclinical inflammation during remission, optimizing steroid dosing to minimize toxicity, and preempting relapses. This approach would transform glycomics into an actionable tool for precision management of BP.

Patients with BP typically exhibit tense blisters and erythema on the trunk and limbs, although a subset of patients may also experience mucosal involvement. Previous research has shown that the BP180 autoantibody can be detected in the saliva of individuals with mucous membrane pemphigoid. In our study, we confirmed that 60% of BP patients have a positive antibody titer for BP180 in their saliva, indicating that salivary autoantibodies offer a diagnostic value comparable to that of serum samples. Saliva has emerged as a valuable biofluid for non-invasive biomarker discovery in various diseases. Sialic acid residues have been found in salivary mucins, and a reduction in their levels may lead to mucin structure destabilization and reduced protection against microbial infections (22). Building upon this knowledge, we investigated the abnormal glycosylation structures in salivary proteins of BP patients. We observed a significant increase of sialic acid and fucose glycosylation recognized by SNA, SBA, and AAL in BP patients’ salivary proteins, which were correlated with disease severity. Further correlation analysis has revealed a negative correlation between the level of sialic acid in saliva and the antibody titer, which is consistent with the correlation findings observed in serum. Additionally, ROC curve analysis indicates that sialic acid in saliva and serum consistently demonstrates good discriminatory power. These results suggest that salivary sialic acid could potentially serve as a non-invasive and safe biomarker, providing an alternative to serum in reflecting the severity of the disease.

Our research has certain limitations that should be acknowledged. Firstly, the rarity of BP poses significant challenges in rapidly accumulating a substantial number of patient samples, thus limiting the sample size of our study. To address this, future endeavors should strive to incorporate larger sample cohorts to enhance the precision and robustness of our findings. Secondly, the intricate and multifaceted nature of glycosylation processes complicates the precise delineation of their specific biological contributions. Consequently, any conclusions drawn from observed abnormal glycosylation patterns in the pathology of BP must be approached with due caution. Future research should endeavor to elucidate the precise molecular mechanisms underlying these glycosylation aberrations in the context of BP. Finally, the design of additional diagnostic experiments to assess the specificity and sensitivity of the identified glycosylation markers holds immense potential. Such endeavors would aid in optimizing their clinical applicability as diagnostic tools for BP, further advancing our understanding and treatment of this complex disease.

Our research provides compelling evidence demonstrating the involvement of glycosylation abnormalities in the pathological mechanisms underlying BP. We have successfully identified key abnormal glycosylation structures in serum, blister fluid, and saliva, shedding light on their potential significance in the diagnosis and management of the disease. Notably, our findings establish a strong correlation between these abnormal glycosylation changes and clinical indicators, thereby positioning them as promising diagnostic biomarkers for BP. Specifically, we observed significant alterations in sialic acid and fucose glycosylation within salivary proteins, which closely paralleled disease progression and could serve as reliable markers for assessing disease severity. Understanding the underlying mechanisms of glycosylation changes in BP holds promise in advancing our knowledge of disease pathogenesis and identifying novel therapeutic targets. Continued research in this area is warranted to enhance our understanding of the complex interplay between glycosylation modifications and the development and progression of BP.

## Data Availability

The original contributions presented in the study are included in the article/**Supplementary Material**. Further inquiries can be directed to the corresponding authors.
