# Supplementary material for: Aberrant glycosylation patterns as potential biomarkers for diagnosis and disease progression in bullous pemphigoid
Source: Front Immunol. 2025 May 26;16:1538126. doi: 10.3389/fimmu.2025.1538126 (PMC12146333; doi:10.3389/fimmu.2025.1538126)
Supplement: Supplementary file 1 [file DataSheet1.docx]

Supplementary Material

# 1 Supplementary Tables

**Supplementary Table 1: The treatment processes of the patients corresponding to the saliva samples**

| **Id** | **Therapeutic agents** | **The highest dose of glucocorticoids after admission** | **The glucocorticoid dosage at discharge** | **Length of hospital stay** |
| --- | --- | --- | --- | --- |
| Saliva_BP_1 | Methylprednisolone | 8mg | 8mg | / |
| Saliva_BP_2 | Methylprednisolone | 40mg | 20mg | 12 |
| Saliva_BP_3 | Methylprednisolone | 24mg | 24mg | 8 |
| Saliva_BP_4 | Halometasone Cream | / | / | / |
| Saliva_BP_5 | Methylprednisolone | 20mg | 16mg | 12 |
| Saliva_BP_6 | Methylprednisolone | 40mg | 32mg | 8 |
| Saliva_BP_7 | Methylprednisolone | 4mg | 4mg | / |
| Saliva_BP_8 | Methylprednisolone | 16mg | 16mg | / |
| Saliva_BP_9 | Methylprednisolone | 60mg | 40mg | 12 |
| Saliva_BP_10 | Methylprednisolone | 40mg | 20mg | 5 |
| Saliva_BP_11 | Methylprednisolone | 30mg | 32mg | 9 |
| Saliva_BP_12 | Methylprednisolone | 20mg | 20mg | 11 |
| Saliva_BP_13 | Methylprednisolone | 40mg | 20mg | 11 |
| Saliva_BP_14 | Methylprednisolone | 60mg | 32mg | 10 |
| Saliva_BP_15 | Methylprednisolone | 40mg | 32mg | 8 |
| Saliva_BP_16 | Methylprednisolone | 80mg | 60mg | 20 |
| Saliva_BP_17 | Methylprednisolone | 80mg | 32mg | 14 |
| Saliva_BP_18 | / | / | / | / |
| Saliva_BP_19 | Methylprednisolone Dupilumab | 80mg | / | 7 |
| Saliva_BP_20 | Methylprednisolone Mycophenolate Mofetil | 60mg | / | 5 |

**Supplementary Table 2:** **Sugar-binding specificities of the lectins**

| **Lectin** | **Specificity** | **Print monosaccharide** |
| --- | --- | --- |
|  |  |  |
| Jacalin | Galβ1-3GalNAcα-Ser/Thr(T), GalNAcα-Ser/Thr(Tn), GlcNAcβ1-3-GalNAcα-Ser/Thr(Core3), sialyl-T(ST). not bind to Core2, Core6, and sialyl-Tn (STn) | Galactose |
| ECA | Galβ-1,4GlcNAc (type II), Galβ1-3GlcNAc (type I) | Galactose |
| HHL | High-Man, Manα1-3Man, Manα1-6Man, Man5-GlcNAc2-Asn | Mannose |
| WFA | Terminal with GalNAcα/β1-3/6Gal | GalNAc |
| GSL-II | GlcNAc and agalactosylated tri/tetra antennary glycans | GlcNAc |
| MAL-II | Siaα2-3Galβ1-3GalNAc, Siaα2-3Galβ1-4Glc(NAc)/Glc, Siaα2-3Gal, Siaα2-3, Siaα2-3GalNAc |  |
| PHA-E | Bisecting GlcNAc, biantennary complex-type N-glycan with outer Gal | GlcNAc |
| PTL-I | GalNAc, GalNAcα-1,3Gal, GalNAcα-1,3Galβ-1,3/4Glc | GalNAc |
| SJA | Terminal with GalNAc and Gal, anti-A and anti-B human blood group | GalNAc |
| PNA | Galβ1-3GalNAcα-Ser/Thr(T) | Galactose |
| EEL | Galα1-3(Fucα1-2)Gal (blood group B antigen) | Galactose |
| AAL | Fucα1-6 GlcNAc(core fucose), Fucα1-3(Galβ1-4)GlcNAc | Fucose |
| LTL | Fucα1-2Galβ1-4GlcNAc, Fucα1-3(Galβ1-4)GlcNAc, anti-H blood group specificity | Fucose |
| MPL | Galβ1-3GalNAc, GalNAc | GalNAc |
| LEL | (GlcNAc)n, high mannose-type N-glycans | LacNAc |
| GSL-I | αGalNAc, αGal, anti-A and B | GalNAc |
| DBA | αGalNAc, Tn antigen, GalNAcα1-3((Fucα1-2))Gal (blood group A antigen) | GalNAc |
| LCA | α-D-Man, Fucα1-6GlcNAc, α-D-Glc | Mannose |
| STL | trimers and tetramers of GlcNAc, core (GlcNAc) of N-glycan, oligosaccharide containing GlcNAc and MurNAc | GlcNAc |
| PTL-II | Gal, blood group H, T-antigen | Galactose |
| DSA | β-D-GlcNAc, (GlcNAcβ1-4)n, Galβ1-4GlcNAc | GlcNAc |
| VVA | terminal GalNAc, GalNAcα-Ser/Thr(Tn), GalNAcα1-3Gal | GalNAc |
| MAL-I | Siaα2-3Galβ1-4GlcNAc, Galβ-1,4GlcNAc, Siaα2-3Gal, Galβ1-3GlcNAc, Siaα2-3 | Galactose |
| GNA | High-Mannose, Manα1-3Man | Mannose |
| NPA | High-Mannose, Manα1-6Man | Mannose |
| ACA | Galβ1-3GalNAcα-Ser/Thr (T antigen), sialyl-T(ST) tissue staining patterns are markedly different than those obtained with either PNA or Jacalin | Galactose |
| BPL | Galβ1-3GalNAc, Terminal GalNAc | Galactose |
| PHA-E+L | Bisecting GlcNAc, bi-antennary N-glycans, tri- and tetra-antennary complex-type N-glycan | GlcNAc |
| SNA | Sia2-6Gal/GalNAc | GlcNAc |
| RCA120 | β-Gal, Galβ-1,4GlcNAc (type II), Galβ1-3GlcNAc (type I) | Galactose |
| BS-I | α-Gal, α-GalNAc, Galα-1,3Gal, Galα-1,6Glc | Galactose |
| PSA | α-D-Man, Fucα-1,6GlcNAc, α-D-Glc | Fucose |
| SBA | aα- or β-linked terminal GalNAc, (GalNAc)n, GalNAcα1-3Gal, blood-group A | GalNAc |
| WGA | Multivalent Sia and (GlcNAc)_n_ | GlcNAc |
| UEA-I | Fucα1-2Galβ1-4Glc(NAc) | Fucose |
| PWM | Branched (LacNAc)_n_ | GlcNAc |
| ConA | High-Mannose, Manα1-6(Manα1-3)Man, terminal GlcNAc | Mannose |

**Supplementary Table 3: Basic information of lectin microarray assays samples**

| **Sample ID** | **Gender** | **Age** | **Sample Type** |
| --- | --- | --- | --- |
| Serum_BP1 | Female | 74 | Serum |
| Serum_BP2 | Male | 63 | Serum |
| Serum_BP3 | Female | 74 | Serum |
| Serum_HC1 | Male | 59 | Serum |
| Serum_HC2 | Female | 62 | Serum |
| Serum_HC3 | Female | 68 | Serum |
| Blister_BP1 | Male | 69 | Blister Fluid |
| Blister_BP2 | Female | 64 | Blister Fluid |
| Blister_BP3 | Female | 74 | Blister Fluid |
| Blister_HC1 | Female | 68 | Blister Fluid |
| Blister_HC2 | Male | 59 | Blister Fluid |
| Blister_HC3 | Female | 67 | Blister Fluid |
| Saliva_BP1 | Male | 63 | Saliva |
| Saliva_BP2 | Male | 69 | Saliva |
| Saliva_BP3 | Female | 50 | Saliva |
| Saliva_HC1 | Male | 60 | Saliva |
| Saliva_HC2 | Female | 56 | Saliva |
| Saliva_HC3 | Male | 62 | Saliva |

**Supplementary Table 4: Serum lectin ELISA sample information**

|  | **HC** | **BP** | **Total** |
| --- | --- | --- | --- |
|  | (N=30) | (N=30) | (N=60) |
| **Age** |  |  |  |
| Mean (SD) | 56.0 (5.87) | 63.9 (16.1) | 60.0 (12.6) |
| **Gender** |  |  |  |
| Male | 18 (60.0%) | 18 (60.0%) | 36 (60.0%) |
| Female | 12 (40.0%) | 12 (40.0%) | 24 (40.0%) |

**Supplementary Table 5: Blister fluid lectin ELISA sample information**

|  | **HC** | **BP** | **Total** |
| --- | --- | --- | --- |
|  | (N=13) | (N=29) | (N=42) |
| **Age** |  |  |  |
| Mean (SD) | 64.0 (5.94) | 64.6 (5.43) | 64.4 (5.53) |
| **Gender** |  |  |  |
| Male | 6 (46.2%) | 16 (55.2%) | 22 (52.4%) |
| Female | 7 (53.8%) | 13 (44.8%) | 20 (47.6%) |

**Supplementary Table 6: Saliva lectin ELISA sample information**

|  | **HC** | **BP** | **Total** |
| --- | --- | --- | --- |
|  | (N=20) | (N=20) | (N=40) |
| **Age** |  |  |  |
| Mean (SD) | 67.4 (8.49) | 70.7 (8.03) | 69.0 (8.32) |
| **Gender** |  |  |  |
| Male | 12 (60.0%) | 15 (75.0%) | 27 (67.5%) |
| Female | 8 (40.0%) | 5 (25.0%) | 13 (32.5%) |

**Supplementary Table 7: Measurement of BP180 antibody titers in the saliva of BP patients**

| **ID** | **OD450** | **ID** | **OD450** |
| --- | --- | --- | --- |
| b1 | 0.2449 | b11 | 0.1046 |
| b2 | 0.0621 | b12 | 0.0865 |
| b3 | 0.0603 | b13 | 0.0979 |
| b4 | 0.0521 | b14 | 0.0763 |
| b5 | 0.0558 | b15 | 0.0708 |
| b6 | 0.1018 | b16 | 0.1320 |
| b7 | 0.0700 | b17 | 0.0656 |
| b8 | 0.0599 | b18 | 0.0902 |
| b9 | 0.0623 | b19 | 0.0898 |
| b10 | 0.0519 | b20 | 0.6822 |
| Negative1 | 0.0660 | Negative2 | 0.0644 |
| Positive1 | 1.3638 | Positive2 | 1.3448 |

**Supplementary Table 8: Clinical data information for BP patients in correlation analysis**

| **Variables** | **Serum** | **Blister** | **Saliva** |
| --- | --- | --- | --- |
| **BP180 antibody titer(U/ml)** | 97.36 ± 68.02 | 173.71 ± 63.35 | 112.47 ± 77.2 |
| **BP230 antibody titer(U/ml)** | 21.96 ± 44.07 | 11.50 ± 31.85 | 34.94 ± 37.01 |
| **Eosinophil(10e9/L)** | 1.58 ± 3.32 | 2.42 ± 2.07 | / |
| **Eosinophil%** | 0.1 ± 0.17 | 0.18± 0.14 | / |
| **Age** | 63 ± 16 | 70± 13 | / |
| **Total_IgE(U/ml)** | 5229.81 ± 13466.18 | / | / |
| **hs_CRP(mg/L)** | 29.66 ± 43.15 | / | / |
| **ESR(mm/h)** | 24.25 ± 37.88 | / | / |
| **hos_day(day)** | 11.05 ± 5.2 | / | / |
| **WBCl(10e12/L)** | 10.55 ± 4.63 | / | / |
| **Total Protein(g/L)** | / | 58.06 ± 8.26 | / |
| **Na(mmol/L)** | / | 137.74 ± 5.26 | / |
| **K(mmol/L)** | / | 3.98 ± 0.49 | / |
| **Ga(mmol/L)** | / | 2.05 ± 0.16 | / |
| **Erythema score** | / | / | 22 ± 16 |
| **Mucosal score** | / | / | 3.5 ± 4.8 |
| **BPDAI** | / | / | 48 ± 28 |

**Supplementary Table 9: The saliva-related experimental samples were derived from patients with positive serum BP180 antibody titers**

| **Id** | **The titer of BP180NC16A antibodies (U/mL)** | **Id** | **The titer of BP180NC16A antibodies (U/mL)** |
| --- | --- | --- | --- |
| A1 | 18 | A11 | 88 |
| A2 | 100 | A12 | 210 |
| A3 | 125 | A13 | 78 |
| A4 | 2 | A14 | 150 |
| A5 | 11 | A15 | 130 |
| A6 | 63 | A16 | 229 |
| A7 | 10 | A17 | 219 |
| A8 | 149 | A18 | / |
| A9 | 111 | A19 | 217 |
| A10 | 226 | A20 | 231 |

# 2 Supplementary Figure


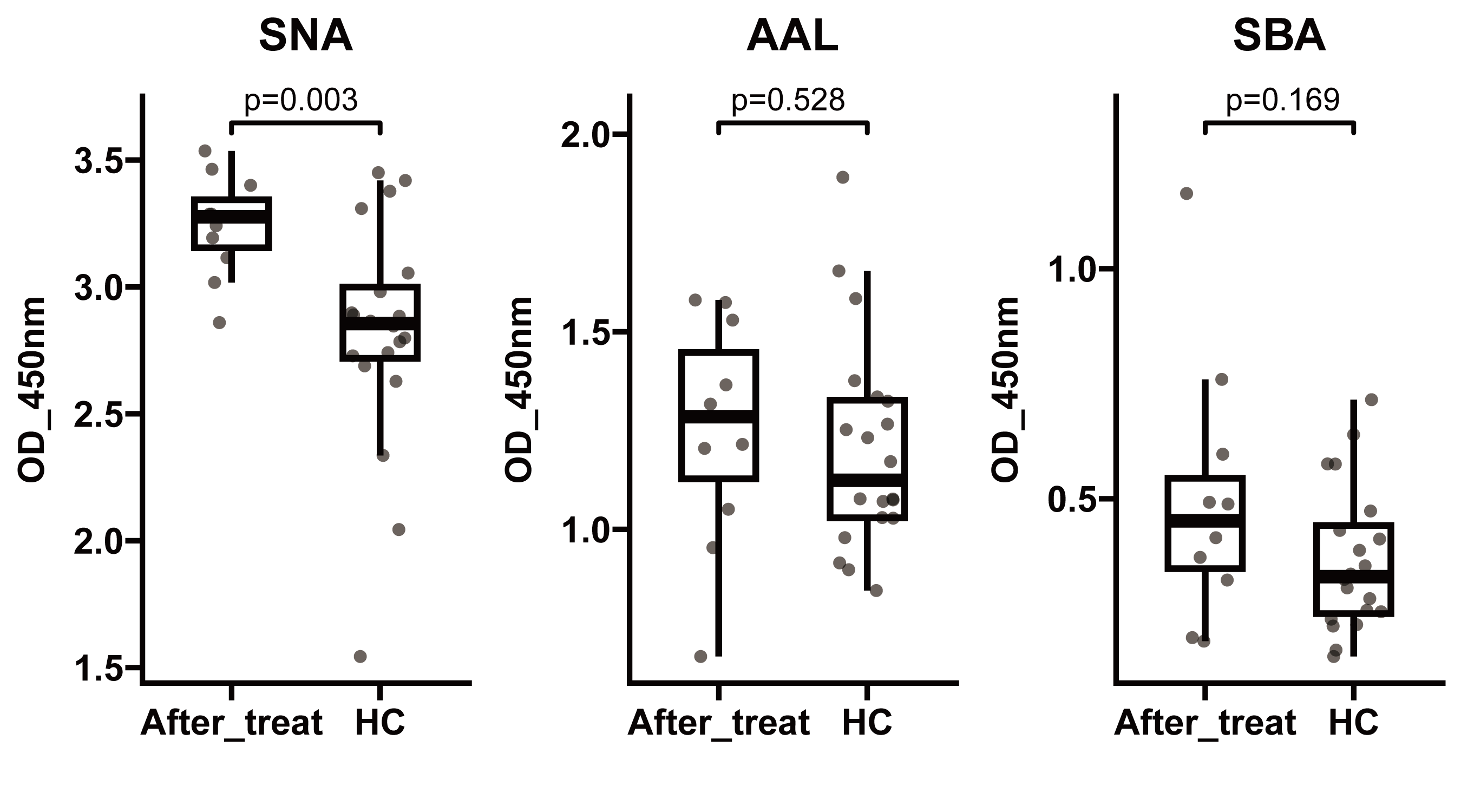


**Supplementary Figure 1:** Comparison of salivary glycan structures binding to SNA and AAL between post-treatment patients and healthy controls. Post-treatment patients (After_treat, n=11) and healthy controls (HC, n=20) were analyzed. Statistical differences were assessed using the Wilcoxon-Mann-Whitney test.
